# Structural and Functional Alterations in Visual Pathway After Optic Neuritis in MOG Antibody Disease: A Comparative Study With AQP4 Seropositive NMOSD

**DOI:** 10.3389/fneur.2021.673472

**Published:** 2021-06-09

**Authors:** Chenyang Gao, Zhizheng Zhuo, Yunyun Duan, Yajun Yao, Lei Su, Xinghu Zhang, Tian Song

**Affiliations:** ^1^Center for Neuroinflammation, Beijing Tiantan Hospital, Capital Medical University, Beijing, China; ^2^Department of Radiology, Beijing Tiantan Hospital, Capital Medical University, Beijing, China; ^3^Department of Neurology, Tianjin Neurological Institute, Tianjin Medical University General Hospital, Tianjin, China

**Keywords:** MOG-ON, AQP4-ON, optic radiation, diffusion tensor imaging, visual acuity, optical coherence tomography

## Abstract

**Background:** Optic neuritis (ON) is an important clinical manifestation of neuromyelitis optic spectrum disease (NMOSD). Myelin oligodendrocyte glycoprotein (MOG) antibody-related and aquaporin 4 (AQP4) antibody-related ON show different disease patterns. The aim of this study was to explore the differences in structure and function of the visual pathway in patients with ON associated with MOG and AQP4 antibodies.

**Methods:** In this prospective study, we recruited 52 subjects at Beijing Tiantan Hospital, including 11 with MOG Ig+ ON (MOG-ON), 13 with AQP4 Ig+ ON (AQP4-ON), and 28 healthy controls (HCs). Fractional anisotropy (FA), mean diffusivity (MD), axial diffusivity (AD), and radial diffusivity (RD) of optic radiation (OR), primary visual cortex volume (V1), brain volume, and visual acuity (VA) were compared among groups. A multiple linear regression was used to explore associations between VA and predicted factors. In addition, we used optical coherence tomography (OCT) to examine thickness of the peripapillary retinal nerve fiber layer (pRNFL) and retinal ganglion cell complex (GCC) in a separate cohort consisting of 15 patients with ON (8 MOG-ON and 7 AQP4-ON) and 28 HCs.

**Results:** Diffusion tensor imaging showed that the FA of OR was lower than controls in patients with AQP4-ON (*p* = 0.001) but not those with MOG-ON (*p* = 0.329) and was significantly different between the latter two groups (*p* = 0.005), while V1 was similar in patients with MOG-ON and AQP4-ON (*p* = 0.122), but was lower than controls in AQP4-ON (*p* = 0.002) but not those with MOG-ON (*p* = 0.210). The VA outcomes were better in MOG-ON than AQP4-ON, and linear regression analysis revealed that VA in MOG-ON and AQP4-ON was both predicted by the FA of OR (standard β = −0.467 and −0.521, *p* = 0.036 and 0.034). Both patients of MOG-ON and AQP4-ON showed neuroaxonal damage in the form of pRNFL and GCC thinning but showed no statistically significant difference (*p* = 0.556, 0.817).

**Conclusion:** The structural integrity of OR in patients with MOG-ON, which is different from the imaging manifestations of AQP4-ON, may be a reason for the better visual outcomes of patients with MOG-ON.

## Introduction

Neuromyelitis optic spectrum disorders (NMOSDs) are autoimmune diseases of the central nervous system whose main symptoms are optic neuritis (ON) and longitudinally extensive transverse myelitis (LETM) (1, 2). Aquaporin-4 antibody (AQP4-ab) has been shown to be a hallmark serological marker of NMOSD (3, 4). In the subgroup of NMOSD patients with negative AQP4-IgG, serum antibody against myelin oligodendrocyte glycoprotein (MOG) has been detected (5). Current research tends to classify MOG-ab related diseases as unique disease entities (6–8).

ON is an inflammation of the optic nerve, manifested as vision loss and pain during eye rotation (9). It is an important clinical manifestation of NMOSD, and it is also an important clinical phenotype related to adult MOG antibody diseases. Many previous studies on the visual pathway of NMOSD patients with ON with AQP4 IgG+ (AQP4-ON) have confirmed anterograde and retrograde synaptic degeneration (10–12). Diffusion tensor imaging (DTI), an advanced magnetic resonance imaging (MRI) technique, has also shown the microstructural damage of visual pathway (10, 11, 13), but research on the NMOSD subgroup of patients after ON with MOG Ig+ (MOG-ON) is limited to conventional MRI (7, 14, 15).

In this context, we analyzed optic radiations based on the probabilistic tractography of DTI, measured visual acuity (VA) to reflect vision, and measured peripapillary retinal nerve fiber layer thickness (pRNFL) and ganglion cell complex (GCC) through optical coherence tomography (OCT). Our research purpose is to further study the differences in visual pathway structure and function between patients with MOG-ON and AQP4-ON.

## Materials and Methods

### Study Objects

**Figure 1:**
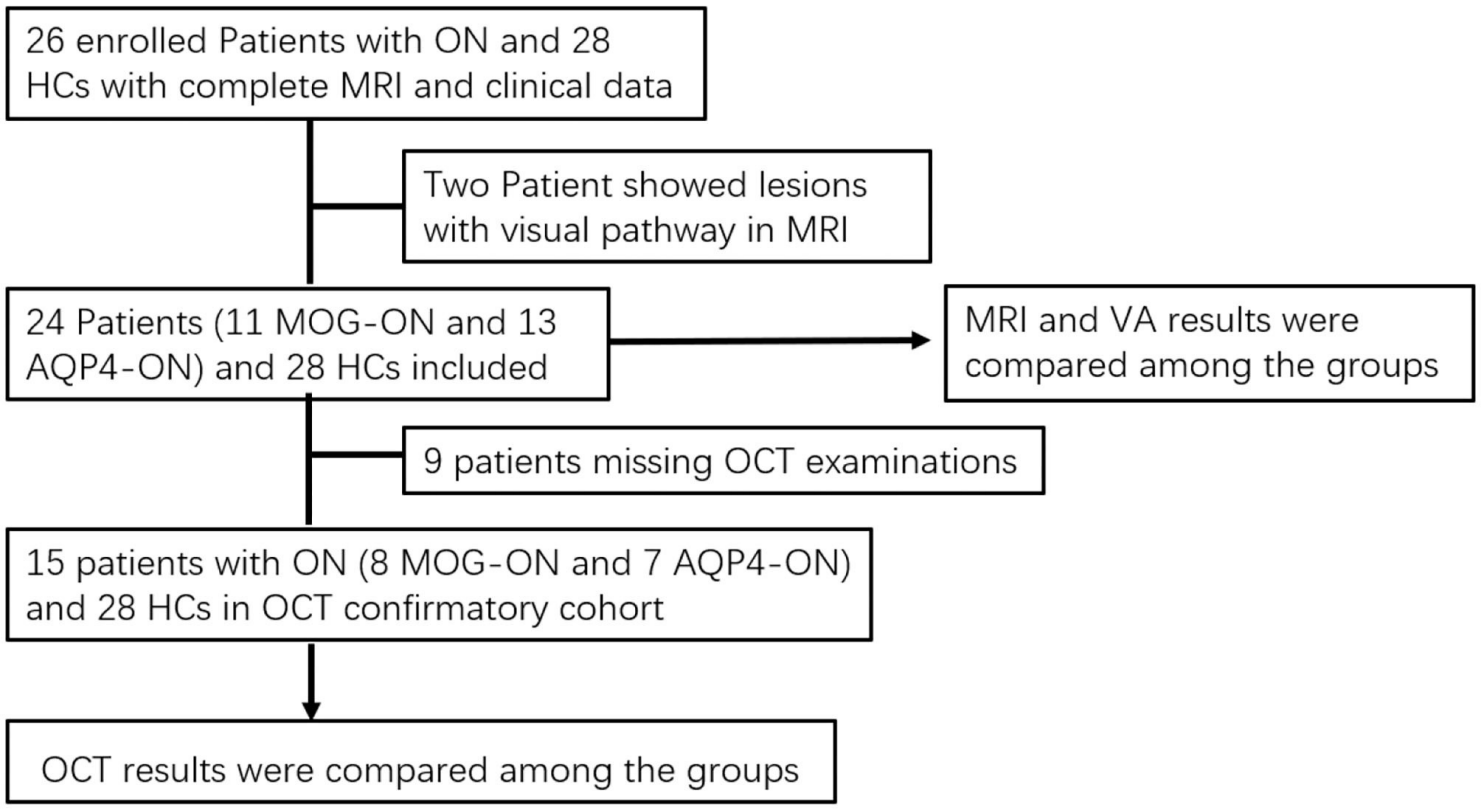
Flowchart of cohort selection. ON, optic neuritis; AQP4, aquaporin-4; MOG, myelin oligodendrocyte glycoprotein; MOG-ON, optic neuritis patients with MOG antibody; MOG-NON, patients with MOG antibody positive without a history of ON; AQP4-ON, NMOSD patients with AQP4 antibody positive and a history of ON; AQP4-NON, NMOSD patients with AQP4 antibody positive but no history of ON; VA, visual acuity; OCT, optical coherence tomography.

As shown in Figure 1, we prospectively analyzed 52 subjects as part of the Clinical and Imaging Patterns of Neuroinflammation Diseases in China (CLUE) trial (National Clinical Trials identifier NCT04106830), including 11 patients with MOG-ON, 13 patients with AQP4-ON, and 28 healthy controls (HCs) admitted from October 2018 to January 2020 at Beijing Tiantan Hospital. The study was conducted in accordance with the Declaration of Helsinki in its currently applicable version and approval by the Medical Ethics Committee of Beijing Tiantan Hospital; all participating patients signed a declaration of written informed consent.

Patients aged 18–80 satisfying the following criteria were eligible for participation in the study: with a diagnosis (by an experienced neurologist) of NMOSD and ON according to the 2015 NMOSD criteria (16); AQP4 or MOG autoantibodies positive based on a cell-based assay (CBA) test; patients with severe visual impairment (VA ≤ 20/200) at the time of the acute phase; and comprehensive head MRI and VA examinations conducted recently at Beijing Tiantan Hospital. Patients who met any of the following criteria were excluded: evidence of OR or V1 lesions on conventional MRI, patients with visual field defects, a history of ON in the last 6 months, pregnancy, human immunodeficiency virus (HIV) or other immunodeficiency syndromes, history of drug abuse (drug or alcohol), and systemic diseases.

Eligible HC participants met the following inclusion criteria: between the ages of 18 and 80, willing to sign an informed consent form, no lesions on conventional MRI, and no other neurological diseases. The exclusion criteria were as follows: pregnancy, eye diseases, and alcohol or drug abuse.

There were several patients in the acute phase of ON (<1 month) in the group that we eliminated in the analysis of OCT data. Finally, OCT had been conducted in 15 of the enrolled patients (8 MOG-ON and 7 AQP4-ON) and 28 HCs. These data were analyzed to compare neuroaxonal damage of the optic nerve among these groups.

### Clinical Evaluation

All patients underwent complete neurological examination and antibody testing based on cytometric bead array. Two experienced neurologists assessed level of disability using the Extended Disability Status Scale (EDSS). Detailed clinical information related to visual symptoms was collected from all patients (Table 1).

**Table 1:** Demographic data of HCs and patients.

		**HC**	**MOG-ON**	**AQP4-ON**
Patients		28	11	13
Age	Mean ± SD	39.64 ± 14.24	41.09 ± 12.87	41.77 ± 10.73
Sex (M/F)		9/19	2/9	2/11
Age at onset	Mean ± SD	N	37.73 ± 14.66	37.69 ± 11.24
ON duration (years)	Mean ± SD	N	3.20 ± 3.71	3.72 ± 4.58
Number of ON episodes	Median (min–max)	N	2 (1–3)	1 (1–4)
EDSS	Median (min–max)	N	3.5 (1–5)	4 (1–8.5)
RTX treatment	Ratio	N	3/11	8/13
AZA treatment	Ratio	N	3/11	4/13

*EDSS, expanded disability status scale; HC, healthy control; MOG-ON, optic neuritis patients with MOG antibody; AQP4-ON, NMOSD patients with AQP4 antibody positive and a history of ON; RTX, rituximab; AZA, Azathioprine*.

### MRI acquisition

MRI scans were conducted on a 3.0-T MR scanner (Philips CX, Best, The Netherlands) including fluid-attenuated inversion recovery imaging (FLAIR) and DTI. Protocols of FLAIR images were as follows: 3D sagittal acquisition by inversion recovery turbo spin echo (IR-TSE), time of repetition (TR)/ time of echo (TE) = 4800 ms/228 ms, inversion recovery = 1650 ms, flip angle = 90°, voxel size = 1 mm × 1 mm × 1 mm, matrix size = 256 × 256, slice number = 196. DTI protocols were as follows: 2D axial acquisition by spin echo-echo planar imaging, TR/TE = 4,000 ms/88 ms, FA = 90°, voxel size = 2.5 mm × 2.5 mm, slice thickness = 2.5 mm, slice gap = 0.25 mm, matrix = 96 × 96, slice number = 60, *b* values = 0 and 1000 s/mm^2^, diffusion gradient direction = 48 (17).

### MRI Processing

The white matter hyperintensity lesions on FLAIR were manually segmented by two experienced neuroradiologists (authors TZ and YD) using 3D Slicer software (https://www.slicer.org/). The 3D T1 image was lesion-filled at the average intensity of surrounding white matter of normal appearance using the Lesion Segmentation Tool for SPM (version 3.0.0, https://www.applied-statistics.de/lst.html) (17). Segmentation of lesion-filled 3D T1 images was conducted using the Computational Anatomy Toolbox in Statistical Parametric Mapping (SPM, version 12 https://www.fil.ion.ucl.ac.uk/spm/). Visual areas were manually outlined on T1 template in Montreal Neurological Institute space and then the corresponding volumes within the outlined areas were obtained.

DTIs were pre-processed using the FMRIB Software Library (FSL version 6.0, https://fsl.fmrib.ox.ac.uk/fsl/fslwiki/FSL) including eddy current and motion correction, skull strip, and DTI parameter fitting. Fractional anisotropy (FA), mean diffusivity, axial diffusivity, and radial diffusivity were obtained. Tract-Based Spatial Statistics (TBSS) was conducted using FSL including non-linear co-registration of individual FA images to a predefined FA template. The mean FA image of all the normalized individual FA images was calculated and used as a basis for extraction of the white matter skeleton. Finally, the individual local maximum FA values were projected onto the mean FA skeleton. Non-FA parameters including mean diffusivity, axial diffusivity, and radial diffusivity were projected onto the mean FA skeleton using the same transformation information obtained in the FA processing. The mean values of all the above DTI parameters within specific atlas-based white matter regions including the posterior thalamic radiation (including OR) were extracted and used for analysis.

### Ophthalmic Examinations

All patients underwent a comprehensive eye examination. The Early Treatment Diabetic Retinopathy Study (ETDRS) scale was used to measure best-corrected distance visual acuity (BCVA) of the patients in the remission period of ON (6 months after onset). For subjects who were unable to read the letters at the viewing distance of 1 m, VA was evaluated using the following four levels in descending order: finger counting ability (CF), hand movement perception ability (HM), light perception ability (LP), or no light perception (NLP). We used the logarithm of the minimum angle of resolution (logMAR) scale of ETDRS VA for statistical analysis (18). For purposes of analysis, logMAR VA values <0.0 were recorded as 0.0, and those >1.0 were recorded as 1.0, resulting in a range of VA (logMAR) from 0.0 (decimal 20/20) to 1.0 (decimal 20/200) (19). Patients with severe visual impairment were defined as having a BCVA (ETDRS) poorer than 20/200 at the time of the acute phase. EDTRS VA of better than 20/40 at the remission phase is considered a good visual outcome (20).

### OCT Image

Spectral domain OCT (Avanti RTVue-XR; Optovue, Fremont, California, USA; software V.2017,1,0,155) was conducted in each patient of the separate cohort by an experienced and certified physician (21). An 840-nm wavelength laser with a tuning range of 100 nm was used to scan at 100 kHz axial frequency. The image resolution was 5.3 mm axially and 18 mm laterally. The optic nerve head map protocol was used to obtain the peripapillary RNFL thickness, and the scanning range covered a circle with a diameter of 3.45 mm centered on the optic disc. The thickness of the GCC was obtained using the GCC scanning protocol, which generates data by instantaneous scanning of 1 mm thickness centered on the fovea and covering a square grid (7 mm × 7 mm) on the central macula. Only high-quality images with signal strength index ≥40 were accepted according to the OSCAR-IB criteria. We report our quantitative OCT data in line with the APOSTEL recommendations (22).

### Statistical Analysis

Data analysis was conducted using SPSS Statistical software version 22 (IBM, Armonk, NY) and graphs were plotted using GraphPad Prism version 8.0 (GraphPad Software, La Jolla, CA). Data are expressed as *n* (%), mean (SD), or median (IQR). Normality of the data was evaluated using the Kolmogorov–Smirnov test and histograms. *P*-value < 0.05 was considered statistically significant. To assess the damage of visual pathway in ON, group differences in MRI and OCT were evaluated by general estimate equation (GEE) models accounting for within-subject intereye dependencies and correcting for age and sex. GEE models were used correcting for age and sex to analyze relationships between structural parameters.

Significantly, the OR and V1 of each hemisphere receive signals from the temporal part of the ipsilateral retina and the nasal part of the contralateral retina. It is important for the analysis of the visual structure from the retina to the visual cortex that the V1 and OR of the left and right hemispheres and the GCC and RNFL of the left and right eyes are calculated separately and then analyzed together, thus doubling the sample size.

Finally, multiple linear regression was adopted to analyze the influence on VA using the average FA of left and right OR, the course of the disease, age of onset, and the number of inflammatory episodes as candidate predictors. For the analysis of visual outcomes, to avoid the bias of inter-relationship between two eyes in the patients with bilateral neuritis, data from only the more severely affected eye were included. Collinearity and interactions between variables were tested before regression analysis and the goodness of fit was evaluated using Pearson's test.

The significance level of all statistical analyses was *p* < 0.05.

## Results

### Patient Characteristics

As shown in Table 1, this study included 24 patients with ON (11 MOG-ON and 13 AQP4-ON) and 28 HCs. The mean ON duration (time since ON) in patients with MOG-ON was 3.20 ± 3.71 years, and that in patients with AQP4-ON was 3.72 ± 4.58 years. The age and gender were well-matched among groups. The median EDSS of patients with MOG-ON was 3.5 and that of patients with AQP4-ON was 4. In the past 12 months, 12 (92.3%) AQP4-ON patients and 6 (54.5%) MOG-ON patients received treatment to improve their disease (rituximab or azathioprine). All patients received oral glucocorticoid therapy.

### MRI Results

**Figure 2:**
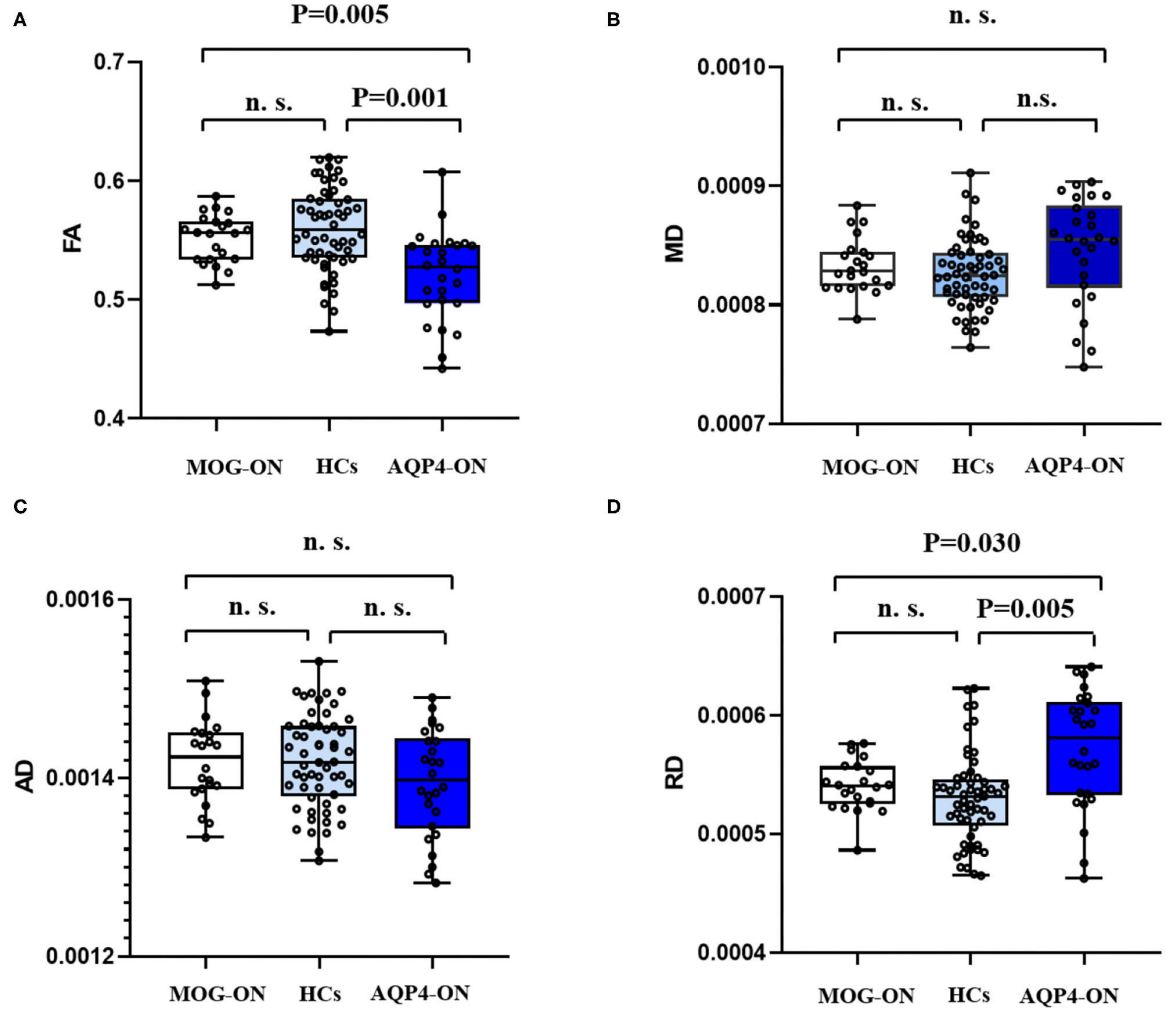
DTI results. Boxplot of mean FA values **(A)**, MD values **(B)**, AD values **(C)**, and RD values **(D)** for posterior thalamic radiation (include OR) in MOG-ON (left, white), HCs (middle, light blue), and AQP4-ON (right, dark blue). FA, fractional anisotropy; MD, mean diffusivity; AD, axial diffusivity; RD, radial diffusivity; HC, healthy control; MOG-ON, optic neuritis patients with MOG antibody; AQP4-ON, NMOSD patients with AQP4 antibody positive and a history of ON; OR, optic neuritis.

The data of MRI results are shown in Table 2. DTI-based probabilistic tractography was used to analyze microstructural white matter changes in OR. Compared with HC, the FA of OR was significantly reduced in patients with AQP4-ON, but not in those with MOG-ON (*p* = 0.001 and *p* = 0.329, respectively) and was significantly lower in AQP4-ON than in MOG-ON (*p* = 0.005) as shown in Figure 2.

**Table 2:** MRI and OCT results from HCs and optic neuritis subgroup.

	**HC**	**MOG-ON**	**AQP4-ON**	**HC vs. MOG-ON**	**HC vs. AQP4-ON**	**MOG-ON vs. AQP4-ON**
V1	2.45 ± 0.32	2.30 ± 0.36	2.07 ± 0.40	0.210	0.002	0.122
Brain volume	1120.12 ± 107.43	1043.41 ± 48.08	1036.40 ± 114.26	0.036	0.016	0.865
FA	5.60 ± 0.35	5.52 ± 0.20	5.21 ± 0.38	0.329	0.001	**0.005**
MD	8.26 ± 0.30	8.33 ± 0.23	8.45 ± 0.45	0.363	0.117	0.322
AD	14.19 ± 0.52	14.18 ± 0.46	13.94 ± 0.60	0.985	0.124	0.182
RD	5.30 ± 0.38	5.54 ± 0.22	5.71 ± 0.49	0.242	0.005	**0.030**
Avg pRNFL(μm)	104.93 ± 6.51	73.13 ± 16.92	79.14 ± 28.35	<0.001	0.003	0.556
Avg GCC (μm)	97.88 ± 5.25	75.19 ± 12.36	76.64 ± 17.05	<0.001	<0.001	0.817

*DTI, diffusion tensor imaging; OCT, optical coherence tomography; V1, Primary visual cortex volume; Brain volume, Whole brain volume including gray matter and white matter; FA, fractional anisotropy; MD, mean diffusivity; AD, axial diffusivity; RD, radial diffusivity; HC, healthy control; MOG-ON, optic neuritis patients with MOG antibody; AQP4-ON, NMOSD patients with AQP4 antibody positive and a history of ON; pRNFL, peripapillary retinal nerve fiber layer thickness; Avg pRNFL, average pRNFL; GCC, retinal ganglion cell complex; Avg GCC, average GCC*.

**Figure 3:**
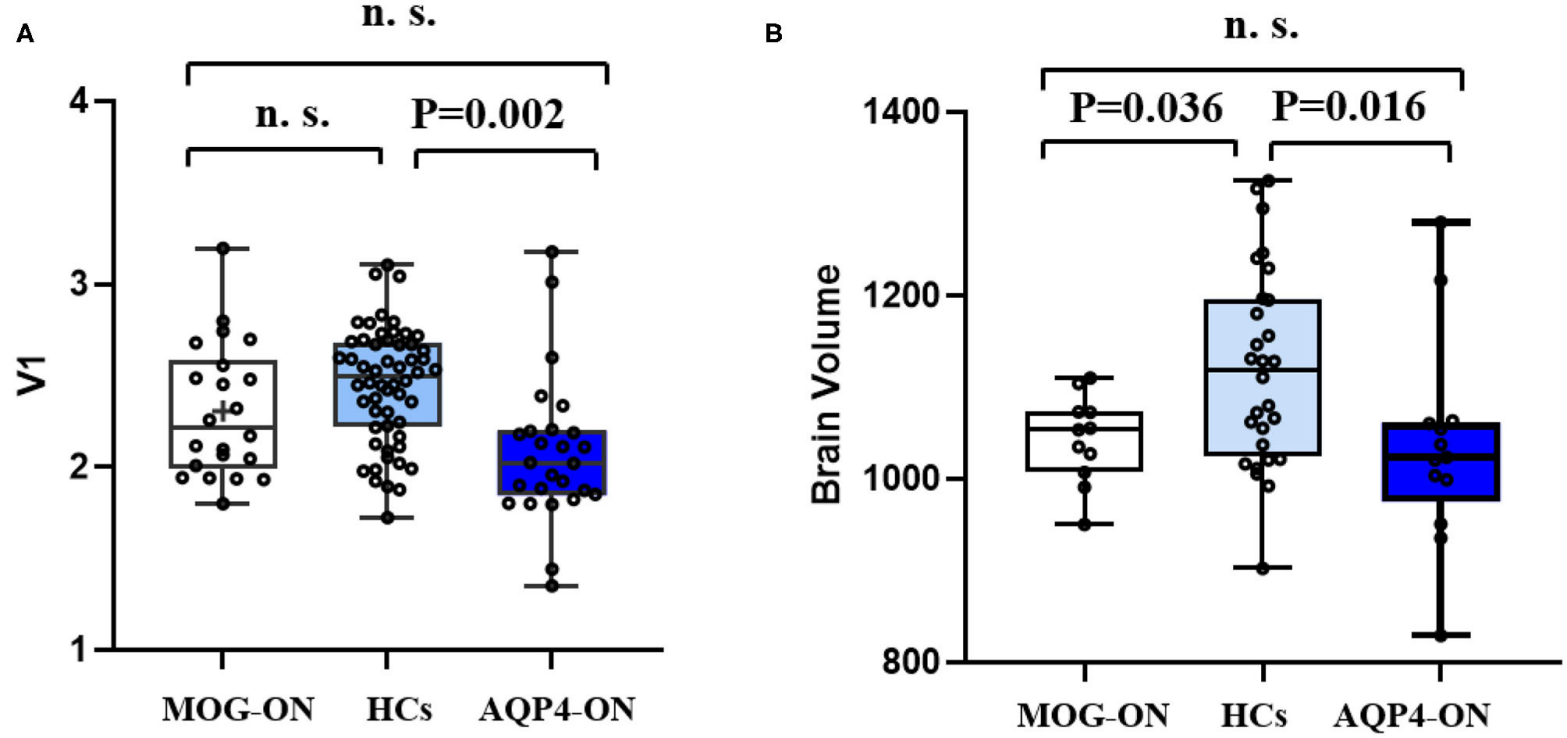
V1 and brain volume in patients and HCs. Boxplot of V1 **(A)** and brain volume **(B)** in MOG-ON (left, white), HCs (middle, light blue), and AQP4-ON (right, dark blue). V1, primary visual cortex volume; brain volume, whole brain volume including gray matter and white matter; HC, healthy control; MOG-ON, optic neuritis patients with MOG antibody; AQP4-ON, NMOSD patients with AQP4 antibody positive and a history of ON; OR, optic neuritis.

Corresponding values in V1 were similar in patients with MOG-ON and AQP4-ON (*p* = 0.122), while the values in V1 were significantly reduced in patients with AQP4-ON (*p* = 0.002) but not in those with MOG-ON (*p* = 0.210). Brain volume was similar in patients with MOG-ON and AQP4-ON (*p* = 0.865) while both were significantly lower than HCs (*p* = 0.036 and 0.016, respectively) (Figure 3).

### Vision Results

**Figure 4:**
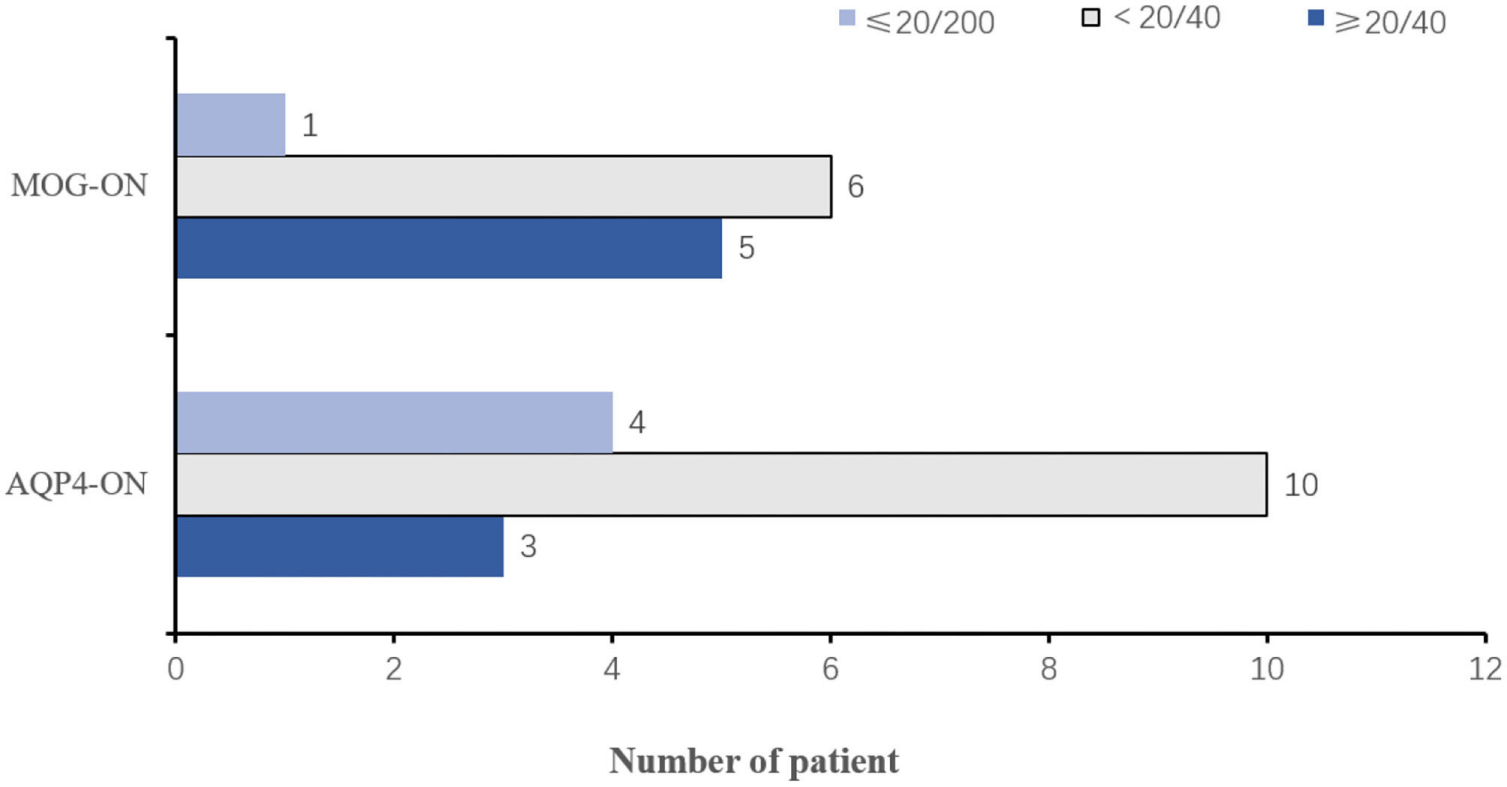
Visual outcomes in MOG-ON and AQP4-ON patients. Histogram of the number of patients with MOG-ON and AQP4-ON in the VA ≤ 20/200 (superior, light blue), VA <20/40 (middle, light gray), and VA ≥ 20/40 (inferior, dark blue) intervals; VA, visual acuity; MOG-ON, optic neuritis patients with MOG antibody; AQP4-ON, NMOSD patients with AQP4 antibody positive and a history of ON; OR, optic neuritis.

Five patients with MOG-ON had bilateral neuritis, while this applied to eight patients with AQP4-ON. EDTRS VA was better than 20/40 in 5 (45.45%) eyes of patients with MOG-ON and 3 (23.08%) with AQP4-ON, while VA was poorer than this level in 7 (54.55%) eyes of patients with MOG-ON (1 classified as NLP) and 10 (76.92%) with AQP4-ON (1 classified as LP, 2 as NLP) (Figure 4). The proportion of MOG-ON patients with good visual outcome was higher than that of AQP4-ON patients, and the proportion of patients with severe visual disability was less than that of AQP4-ON patients (Figure 4).

### VA Outcomes: Correlations With FA and Disease Duration

Covariates such as age, disease duration, number of ON episodes, and FA value of OR were entered into the linear regression model (Table 3). Linear regression analysis showed that VA of patients with MOG-ON and AQP4-ON were both predicted by the FA of OR (standard β = −0.467 and −0.521, respectively; *p* = 0.036 and 0.034, respectively). In MOG-ON only, VA was also predicted by disease duration, age, and number of episode.

**Table 3:** Linear regression of visual acuity with MOG-ON and AQP4-ON.

	**VA in MOG-ON**			**VA in AQP4-ON**		
	**Unstandardized coefficients**		***P*-value**	**Unstandardized coefficients**		***P*-value**
	***B***	**Std. error**		***B***	**Std. error**	
**FA**	−10.981	4.087	**0.036**	−5.017	1.968	**0.034**
**Age**	0.016	0.006	**0.040**	0.011	0.007	0.140
**Disease duration**	0.006	0.002	**0.019**	0.001	0.001	0.362
**Number of episodes**	0.271	0.094	**0.027**	0.132	0.111	0.269

*VA, visual acuity; MOG-ON, optic neuritis patients with MOG antibody; AQP4-ON, NMOSD patients with AQP4 antibody positive and a history of ON; FA, fractional anisotropy*.

### OCT Results

**Figure 5:**
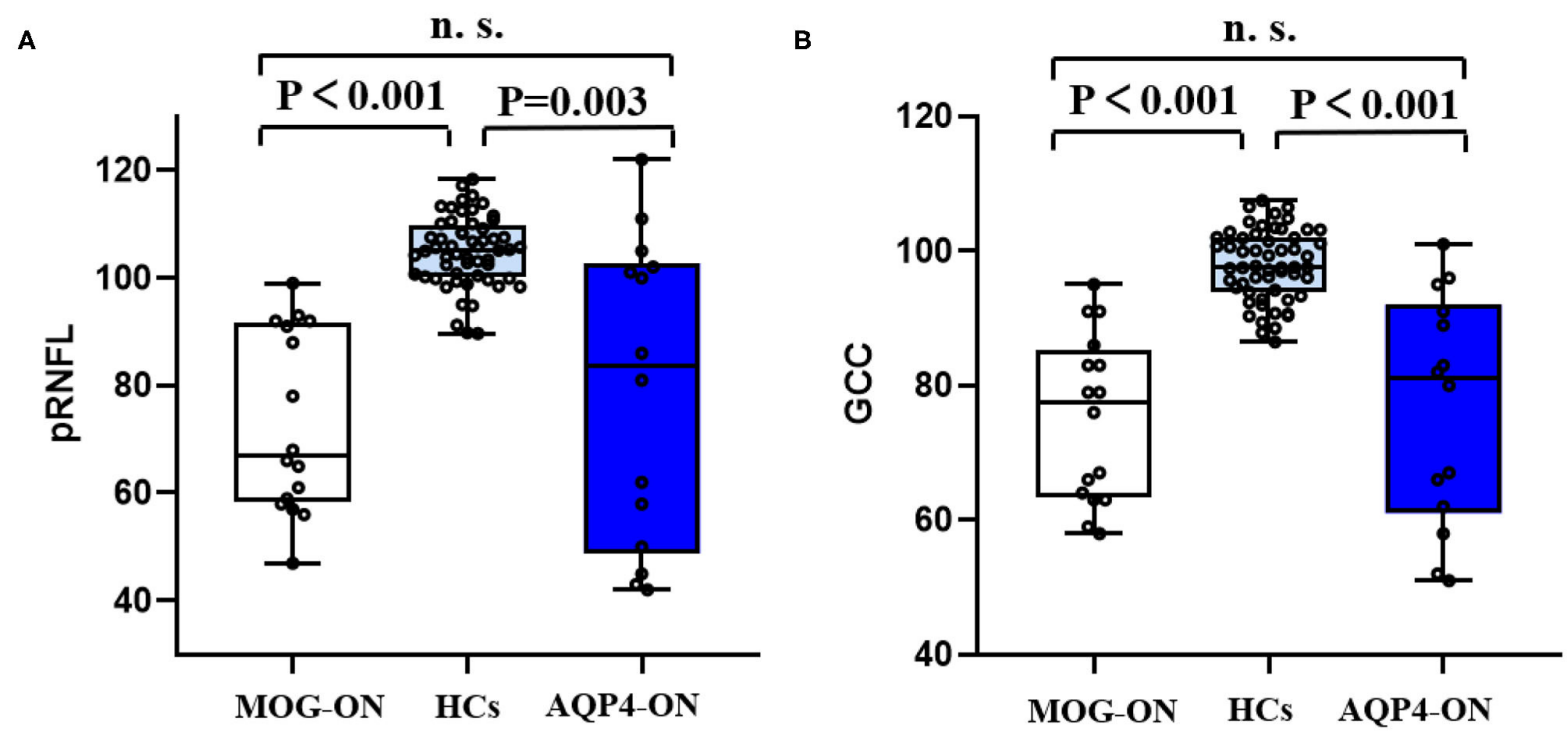
OCT results in patients and HCs. Boxplot of V1 **(A)** and brain volume **(B)** in MOG-ON (left, white), HCs (middle, light blue), and AQP4-ON (right, dark blue). pRNFL, peripapillary retinal nerve fiber layer thickness; GCC, retinal ganglion cell complex; HC, healthy control; MOG-ON, optic neuritis patients with MOG antibody; AQP4-ON, NMOSD patients with AQP4 antibody positive and a history of ON; OR, optic neuritis.

As shown by Table 2, in the separate OCT cohort, the pRNFL and GCC of patients with MOG-ON were significantly less than in those with HCs (*p* < 0.001 in both cases). Similarly, the RNFL and GCC of AQP4-ON patients were significantly lower than in HCs (*p* = 0.003 and *p* < 0.001, respectively). However, the RNFL and GCC of MOG-ON patients and AQP4-ON patients were similar (*p* = 0.556 and *p* = 0.817, respectively) (Figure 5).

## Discussion

The above data from OR and V1 indicate that prior ON may induce anterograde synaptic degeneration of the visual cortex, and the degree of damage in patients with MOG-ON is less than in patients with AQP4-ON.

This study reveals the structural and functional alterations in visual pathways of MOG-ON and AQP4-ON patients. Episodes of ON triggered both anterograde (OR, V1) and retrograde (retinal) axon degeneration. The results showed that in anterograde axon injury, there is no evidence of OR microstructure damage in patients with MOG-ON, while the OR in patients with AQP4-ON showed clear microstructural damage, although the proportion of AQP4-ON patients receiving immunosuppressive therapy is much higher than that of MOG-ON patients. Additionally, the proportion of MOG-ON patients with good visual outcomes was higher than that of AQP4-ON patients, and the VA of patients with MOG-ON and AQP4-ON were both predicted by the FA of OR. This finding suggests that visual outcomes in patients with MOG-ON and AQP4-ON are related to OR integrity.

The visual pathway is a well-defined neural pathway that transmits visual information from the retina to the primary visual cortex. Each OR and V1 receives signals representing the contralateral visual field. We analyzed data from left and right V1 and OR, to assess the corresponding visual pathways (10). This increases statistical power and allows any damage in the visual pathway to be accurately mapped.

One study has shown that MOG-ON leads to severe pRNFL and GCIP thinning, the extent of which is comparable to AQP4-ON (23). Another has shown that MOG-ON patients experience less retinal neuronal loss than AQP4-ON (24). The relapse rate of MOG-ON is obviously higher than that of AQP4-ON (23, 25, 26); the reasons for the inconsistent results of the two studies may be related to the time phase and follow-up time of the enrolled patients. In contrast to AQP4-ON in which damage is caused by the severity of an attack, the damage associated with MOG-ON seems to be driven by the frequency of attacks. We found no significant difference in RNFL or GCC damage between patients with MOG-ON and AQP4-ON, which indicates similar degrees of retinal neuronal damage in both types of ON.

Previous research has shown that patients with MOG-ON have severe vision loss in the acute phase, with better recovery than patients in AQP4-ON, which indicates good visual outcomes in MOG-ON (27). The present study is broadly consistent with this finding.

It is thought that AQP4 Ig+-related diseases may be caused by an increase in astrocytes and that Müller cells as astrocytes specific to the retina express AQP4 antibody (28). The OR and retinal damage in visual pathways of the patients with AQP4-ON has been clearly confirmed in previous studies (29). Microstructural changes have been found in the afferent visual system (OR) of AQP4-ab seropositive NMOSD patients without a history of ON, which indicates that AQP4 antibody-related diseases are associated with the location of AQP4 expression. Müller cell dysfunction plays a major role in the impairment of retinal function and the structural changes sof the visual pathway in AQP4-ON. The structural damage in AQP4-ab-positive-related diseases may be closely related to the accumulation of astrocytes, but the evidence for this in MOG-ON patients is unclear. The present study aimed to understand structure and function of the visual pathway in ON patients, including those with MOG-ON. We found a lack of microstructural damage in the OR of MOG-ON patients, and the VA outcomes were significantly related to OR integrity.

These data suggest that AQP4-ON and MOG-ON have different mechanisms of neurodegeneration. Although both AQP4 and MOG antibody ON are antibody-mediated demyelinating diseases of the central nervous system, and both have pathogenic components of inflammation and neurodegeneration, the severity of the disease and the degree of pathogenic factors may differ between them. In the pathogenesis of AQP4 antibody-mediated ON, abnormal AQP4 dynamics in astrocytes/Müller cells lead to severe neurodegeneration (28) while the pathogenic effect in MOG antibody-positive ON is relatively mild. Compared with AQP4, the lack of AQP4 expressing Müller cell invasion target can only induce increased inflammation and cause tissue damage through indirect effects (5). It was also believed that MOG antibody-related diseases may be associated to more reversible mechanisms compared with AQP4 antibody (30). These differences may explain the lack of OR damage in patients with MOG-ON, and this relative integrity of OR may explain better visual outcomes in this group.

There are some limitations to this exploratory study. Although data reflecting visual pathway structure and function were analyzed, it was a cross-sectional study. In addition, automated analysis of posteriorthalamic radiation using the approach of TBSS to gauge OR damage, but a direct measure of region of interest (ROI) to measure the ROI of OR was not used. Another potential limitation of the study is that the sample size is relatively small. This reflects the fact that MOG antibody-positive ON is a very rare disease with minimal scope for large-scale studies. However, we gathered data from the left and right V1, OR, GCC, and RNFL and combined these for analysis to increase statistical power. This study chose normal VA as the visual outcome may underestimate the actual extent of damage to the afferent visual system. The impact of structural damage should be further studied through low-contrast letter acuity, color vision testing, visual fields, and quality of life scales in the future study.

In summary, this study provides evidence of differential damage to structure and function of the visual pathway in patients with MOG-ON and AQP4-ON. The structural integrity of OR in patients with MOG-ON, which is different from the imaging manifestations of AQP4-ON, may be a reason for the better visual outcomes of patients with MOG-ON.

## Data Availability Statement

The original contributions presented in the study are included in the article/ Supplementary Material, further inquiries can be directed to the corresponding author/s.

## Ethics Statement

The studies involving human participants were reviewed and approved by Beijing Tiantan Hospital Ethics Committee. The patients/participants provided their written informed consent to participate in this study.

## Author Contributions

TS formulated the conception and design of this study. CG, ZZ, YD, YY, and LS contributed to the acquisition and analysis of data, and to critical revisions of the article. TS and CG drafted the article and prepared the figures. All authors contributed to the article and approved the submitted version.

## Conflict of Interest

The authors declare that the research was conducted in the absence of any commercial or financial relationships that could be construed as a potential conflict of interest.
