# Supplementary material for: Structural and Functional Alterations in Visual Pathway After Optic Neuritis in MOG Antibody Disease: A Comparative Study With AQP4 Seropositive NMOSD
Source: Front Neurol. 2021 Jun 9;12:673472. doi: 10.3389/fneur.2021.673472 (PMC8220215; doi:10.3389/fneur.2021.673472)
Supplement: Supplementary file 1 [file Table_1.docx]

Visual acuity and corresponding logMAR value of patients with AQP4-ON.

| Patients ID | Unilateral/ Bilateral | Visual Acuity | logMAR VA |
| --- | --- | --- | --- |
| 1 | Unilateral | 20/100 | 0.7 |
| 2 | Bilateral | 20/20 | 0 |
| 3 | Unilateral | 20/80 | 0.6 |
| 4 | Unilateral | 20/25 | 0.1 |
| 5 | Bilateral | light perception ability | 1 |
| 6 | Bilateral | no light perception | 1 |
| 7 | Bilateral | 20/100 | 0.7 |
| 8 | Bilateral | 20/160 | 0.9 |
| 9 | Unilateral | 20/160 | 0.9 |
| 10 | Bilateral | 20/63 | 0.5 |
| 11 | Bilateral | ＜20/200 | 1 |
| 12 | Bilateral | 20/40 | 0.3 |
| 13 | Unilateral | 失明 | 1 |

Visual acuity and corresponding logMAR value of patients with MOG-ON.

| Patients ID | Unilateral/ Bilateral | Visual Acuity | logMAR VA |
| --- | --- | --- | --- |
| 1 | Unilateral | 20/20 | 0 |
| 2 | Bilateral | 20/125 | 0.8 |
| 3 | Bilateral | 20/160 | 0.9 |
| 4 | Bilateral | 20/20 | 0 |
| 5 | Bilateral | 20/160 | 0.7 |
| 6 | Unilateral | 20/20 | 0 |
| 7 | Bilateral | 20/16 | 0 |
| 8 | Unilateral | 20/25 | 0.1 |
| 9 | Unilateral | 20/50 | 0.4 |
| 10 | Bilateral | no light perception | 1 |
| 11 | Unilateral | 20/160 | 0.9 |
